# Long-Term Environmental Enrichment Relieves Dysfunctional Cognition and Synaptic Protein Levels Induced by Prenatal Inflammation in Older CD-1 Mice

**DOI:** 10.1155/2022/1483101

**Published:** 2022-05-06

**Authors:** Zhe-Zhe Zhang, Li-Ping Zeng, Jing Chen, Yong-Fang Wu, Ya-Tao Wang, Lan Xia, Qi-Gang Yang, Fang Wang, Gui-Hai Chen

**Affiliations:** ^1^Department of Neurology (Sleep Disorders), The Affiliated Chaohu Hospital of Anhui Medical University, Hefei, 238000 Anhui, China; ^2^Department of Neurology, The Second Affiliated Hospital of Anhui Medical University, Hefei, 230601 Anhui, China; ^3^Department of Neurology or Department of Critical Care, The First Affiliated Hospital of Anhui Medical University, Hefei, 230022 Anhui, China

## Abstract

A mounting body of evidence suggests that prenatal inflammation may enhance the rate of age-associated cognitive decline and may involve aberrant amounts of synaptic proteins in the hippocampus, including synaptotagmin-1 (Syt1) and activity-regulated cytoskeleton-associated protein (Arc). However, little is known about the specific impact of adolescent environmental enrichment (EE) on age-associated cognitive decline and the changes in synaptic proteins caused by prenatal inflammation. In this study, CD-1 mice in late pregnancy were given intraperitoneal doses of lipopolysaccharide (LPS, 50 *μ*g/kg) or normal saline. Offspring arising from LPS dams were divided into a LPS group and a LPS plus EE (LPS-E) group. The LPS-E mice were exposed to EE from 2 months of age until the end of the experiment (3 or 15 months old). The Morris water maze (MWM) was used to assess the spatial learning and memory capacities of experimental mice, while western blotting and RNA-scope were used to determine the expression levels of Arc and Syt1 in the hippocampus at the protein and mRNA levels, respectively. Analysis revealed that at 15 months of age, the control mice experienced a reduction in cognitive ability and elevated expression levels of *Arc* and *Syt1* genes when compared to control mice at 3 months of age. The LPS-E group exhibited better cognition and lower protein and mRNA levels of Arc and Syt1 than mice in the LPS group of the same age. However, the enriched environment mitigated but did not counteract, the effects of prenatal inflammation on cognitive and synaptic proteins when tested at either 3 or 15 months of age. Our findings revealed that long-term environmental enrichment improved the expression levels of synaptic proteins in CD-1 mice and that this effect was linked to the dysfunctional cognition caused by prenatal inflammation; this process may also be involved in the reduction of hippocampal *Arc* and *Syt1* gene expression.

## 1. Introduction

The normal aging process is accompanied by a decline in certain cognitive functions, including memory, processing speed, and spatial learning [1, 2]. These age-associated cognitive declines have a negative impact on the quality of life in the elderly. However, the neurological basis of age-associated cognitive decline remains unclear, and there are no effective treatments to slow or reverse the progression of this condition. As a result, coordinated efforts are required to better understand, prevent, and treat age-associated cognitive decline. In previous studies, a rodent model of aging and cognitive decline was used to investigate the expression of synaptic proteins that have been shown to be functionally required for hippocampal plasticity. These studies found that changes in the expression of certain synaptic proteins are associated with aging and may underpin the impaired cognitive performance observed in the elderly, including synaptotagmin-1 (Syt1), postsynaptic density protein 95 (PSD-95), activity-regulated cytoskeleton-associated protein (Arc), Homer1b/c, and the amino-3-hydroxy-5-methyl-4-isoxazolepropionic acid receptor (AMPAR) GluA1 subunit [3–7]. However, much work is still needed to establish a definitive link between changes in synaptic proteins and the decline of cognition during aging.

The administration of lipopolysaccharides (LPS) during pregnancy can create a well-documented and widely accepted mouse model of maternal gestational infection [8]. This treatment activates inflammatory cells and results in higher expression levels of certain proinflammatory cytokines, including interleukin-1*β*, interleukin-6, and tumor necrosis factor-*α*. Maternal inflammation causes abnormalities in brain development that are associated with subsequent cognitive impairment in the offspring. Research with rodent models has demonstrated that experimental inflammation during embryonic development can impair behavioral and cognitive performance in adulthood [9, 10]. The prenatal alteration of microglial function, including inflammation, has been shown to induce synaptic dysfunction in adults that can severely impair learning, memory, and other essential cognitive functions [11, 12]. Our previous studies indicated that the exposure of CD-1 female mice to LPS induces inflammation during late pregnancy, accelerates age-associated cognitive decline and exacerbates age-related changes in the levels of synaptic plasticity-related proteins (including Syntaxin-1, Syt1, Arc, PSD-95, GluA1, and Homer-1b/c) in both the mother and their offspring [13–16].

The provision of an enriched environment (EE) can be used as a noninvasive approach to counteract many of the age-related alterations in cognitive function and hippocampal structure. The provision of an EE can exert several beneficial effects on brain plasticity and function, including remodeling or increased numbers of dendritic branches and spines in several brain structures [2, 17, 18]. In the hippocampus of adult mice, the use of an EE has been demonstrated to facilitate cell proliferation and neurogenesis in the dentate gyrus [19, 20]. Singhal et al. showed that short-term EE (4 weeks) improved the retention of spatial learning during late-to-middle age and that EE had no effect on gene expression in young and middle-aged mice [21]. In a recent study, we demonstrated that long-term postpartum EE alleviated age-associated cognitive decline and a decline in the hippocampal expression of synaptic plasticity-related proteins (including PSD-95, GluA1, and Homer-1b/c) induced by prenatal inflammation in CD-1 mice [16].

Arc is known to mediate a critical period for spatial learning and hippocampal networks [22] and has been widely used as a marker of neurons activated by specific behaviors that are particularly involved in memory consolidation and synaptic plasticity [23, 24]. However, the results of research on Arc are inconsistent. Singh et al. found that the expression of the *Arc* gene was downregulated in elderly mice [3]. However, increased levels of Arc protein are known to be associated with reduced functionality in the ubiquitin-proteasome system that is critical for synaptic plasticity following the activation of memory [25]. The hippocampus contains the highest levels of Syt1, a protein of the presynaptic active zone that is essential for the maintenance of intact synaptic transmission and cognitive function [26]. In another study, de Jong et al. found that the phosphorylation of Syt1 acts downstream of vesicle priming to control synaptic plasticity [27]. However, although we have shown that the provision of EE from adolescence is beneficial for age-associated cognitive decline [28], few studies have investigated whether EE can affect the expression of the synaptic proteins Arc and Syt1 in the hippocampus induced by prenatal inflammation.

In this study, we extended our previous study and investigated whether prenatal exposure to inflammation, either with or without EE from adolescence, affected spatial learning and memory in young (3 months of age) or aged (15 months of age) CD-1 mice. We also evaluated whether the protein and mRNA levels of Syt1 and Arc were altered in the hippocampus of experimental mice of different ages under specific treatment conditions. Finally, we determined the correlations between spatial learning and memory and key neurobiological indicators in different age groups of mice undergoing specific treatments. Our aim was to determine whether the provision of EE from adolescence can alleviate the changes of synaptic proteins in age-associated cognitive decline that are induced by age and prenatal inflammation.

## 2. Materials and Methods

### 2.1. Animal

**Figure 1:**
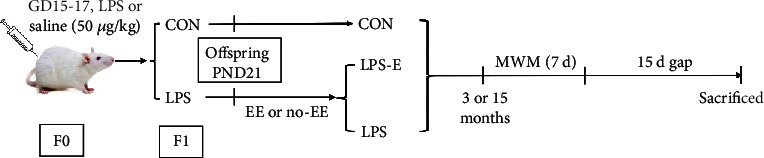
The timeline of experimental events. GD15-17: gestation 15-17 day; CON: untreated control group; LPS: lipopolysaccharide treatment group; E: group of mice exposed to EE; MWM: Morris water maze.

All CD-1mice (6-8 weeks of age) were purchased from the Animal Experimental Center of Anhui Medical University. Mice were housed for two weeks in standard cages (30 × 16 × 11 cm^3^) under a 12 h light/dark cycle in a temperature and humidity-controlled room and were provided with food and water *ad libitum*. Cages were cleaned every 12˗14 days. Two-month-old males and females, weighing 22-25 g, were mated in a ratio of 1 : 2; pregnant mice were kept individually in a single standard cage and recorded as gestation day 0 (GD0). Following normal birth and breastfeeding, the offspring were separated from their mothers on postnatal day 21. Male mice derived from mothers receiving LPS were assigned into the LPS groups that were, respectively, given additional EE (LPS-E) or no EE (LPS) treatment (*n* = 10 per group). Mice derived from the mothers receiving normal saline were assigned into control (CON) groups (*n* = 10). At 3 months-of-age and 15 months of age, all mice underwent behavioral tests, western blotting, and RNA-scope analysis. All animal experiments were carried out in compliance with the guidelines for humane treatment set by the Association of Laboratory Animal Sciences and the Center for Laboratory Animal Sciences at Anhui Medical University (Reference: LLSC20160165).  Figure 1 shows a schematic representation of the experimental timeline.

### 2.2. Drug Administration

Pregnant mice were intraperitoneally injected with LPS (50 *μ*g/kg) or an equal volume of normal saline at GD15-17.

### 2.3. Environment Enrichment

EE animals were housed together in a group of eight in a large cage (36 × 23 × 18 cm^3^) containing an assortment of objects, including climbing ladders, a running wheel, a ball, plastic, and wooden objects suspended from the cage top, paper, cardboard boxes, and nesting material, until the behavioral experiment was complete. Toys were changed every 1–2 days. Control groups were housed in standard-sized cages containing no objects.

### 2.4. Morris Water Maze Test

The Morris water maze (MWM) was performed as described previously [29]. In brief, the MWM consisted of a round opaque tank (150 cm in diameter and 30 cm in height) containing water (21–22°C; depth: 25 cm). A cylindrical platform (10 cm in diameter and 24 cm in height) was fixed in the center of a quadrant in the pool to facilitate mouse escape. In the positioning navigation phase (the learning phase), mice were allowed to search for the platform for 60 s or until they landed on it. If the mice failed to find the platform within 60 s, they were guided to rest on the platform for 30 s. All mice were tested 4 times with a 15 min interval every day for 7 days before being removed back to their holding cage. On the last day (day 7), the platform was removed from the tank after positioning navigation trails, and a probe trial (the memory phase) was conducted (60 s). We recorded the escape latency, swimming distance, swimming speed, and the time spent in each quadrant, using a camera system installed directly above the tank.

### 2.5. Tissue and Serum Preparation

All mice were returned to their original cages after completing the MWM test (without any further stress) and were sacrificed 15 days later for blood and tissue sampling. Brain tissue was quickly removed and stored in a refrigerator for RNA-scope and western blotting.

Whole blood was collected via the eyeball. Serum was prepared by centrifuging blood samples for 5 min at 4000 rpm (4°C). Approximately 100 *μ*l of serum was collected from each mouse and analyzed for corticosterone (CORT) levels with a quantitative ELISA (Demeditec Corticosterone rat/mouse ELISA, Demeditec Diagnostics, Germany). The MAP Mouse Cytokine/Chemokine Magnetic Bead Panel (Millipore, USA) was used to determine the levels of TNF-*α* and IL-1*β*.

### 2.6. Western Blotting

Western blotting was performed as described previously described [15]. In brief, the protein from the right hippocampus of brain tissue was separated by 10% SDS-polyacrylamide gel electrophoresis and then transferred to nitrocellulose membranes. These membranes were then incubated overnight with primary antibodies to rabbit anti-Arc polyclonal antibody (1 : 500, Proteintech, 16290-1-AP) and rabbit anti-Syt1 (1 : 1000, Sigma, S2177) at 4°C. The following morning, the membranes were incubated with a horseradish peroxidase- (HRP-) conjugated anti-rabbit IgG (Zhongshan-Golden Bridge, Beijing, China; 1/10000, 1/10000, respectively) for 2 h at room temperature. Immunoreactive bands were detected and quantified by Image-Pro Plus 6.0 software (Media Cybernetics, USA).

### 2.7. RNA-Scope

The protocol used for RNA-scope is described in our previous paper [14, 27]. In brief, tissue sections and all necessary materials and reagents were prepared before the experiment. First, we heated and boiled 1 × RNAscope Target Retrieval Reagent (ACDBio, cat. no. 322000) in a moist environment. Next, we added RNA-scope H_2_O_2_ and incubated the mixture for 10 minutes. Then, slides were immersed in 1 × RNAscope Target Retrieval and then 100% ethanol; then, the slides were dried. Next, RNA-Scope protease Plus reagent (ACDBio, cat no. 322381) was added to the slides which were then placed in a HybEZ™ hybridization oven and incubated at 40°C. Next, Arc (ACD Bio, cat no. 316911) or Syt1 (ACDBio, cat. no. 491831) mRNA probe mixtures were added and incubated at 40°C for 2 hours. Subsequently, we added RNA target multichannel Fluorescent AMP1 (included in the Multiplex Fluorescent Kit) and incubated at 40°C. AMP2 and AMP3 were added dropwise at 40°C. HRP-C was then added to the slide and incubated at 40°C. Next, 150-200 *μ*L of TSA plus fluorescent dye was added and incubated in a HybEZ™ hybridization oven. Then, RNA-scope multichannel fluorescent second-generation HRP blocker was added and incubated at 40°C. Finally, DAPI was added and incubated for 30 seconds. The DAPI was then removed, and Prolong Gold antiquenching seal was added. Finally, the sections were observed under a fluorescence microscope.

### 2.8. Morphometric Analysis and Quantification

Images of the hippocampus subregions (cornu ammonis [CA]1, CA3, and dentate gyrus [DG]) were captured at (×40) and (×200) magnifications using an Olympus IX71 fluorescent microscope (Olympus, Tokyo, Japan) equipped with a PXL37 CCD camera (Photometrics, Tucson, AZ, United States). RNA-scope hybridization fluorescence was imaged using a ×40 objective on a laser-scanning confocal microscope (Zeiss LSM700 or LSM780). The densitometric analysis of immunoreactivity bands was conducted using the ImageJ software (Media Cybernetics, USA) to calculate the relative expression, so the relative protein level was represented by the average optical density of the immune band. The relative mRNA level was calculated as the number of positive cells expressing mRNA.

### 2.9. Statistical Analysis

All results are expressed as means ± standard error of the mean (S.E.M.). Datasets were analyzed using univariate or repeated measures analysis of variance (ANOVA), followed by least statistical difference (LSD) post hoc tests. Pearson's correlation test was used to determine correlations between the hippocampal levels of Arc and Syt-1 proteins and mRNAs and performance in the MWM. Significance was assumed at *P* < 0.05. All analyses were performed with SPSS 21.0 for Windows.

## 3. Results

### 3.1. MWM Performance

**Figure 2:**
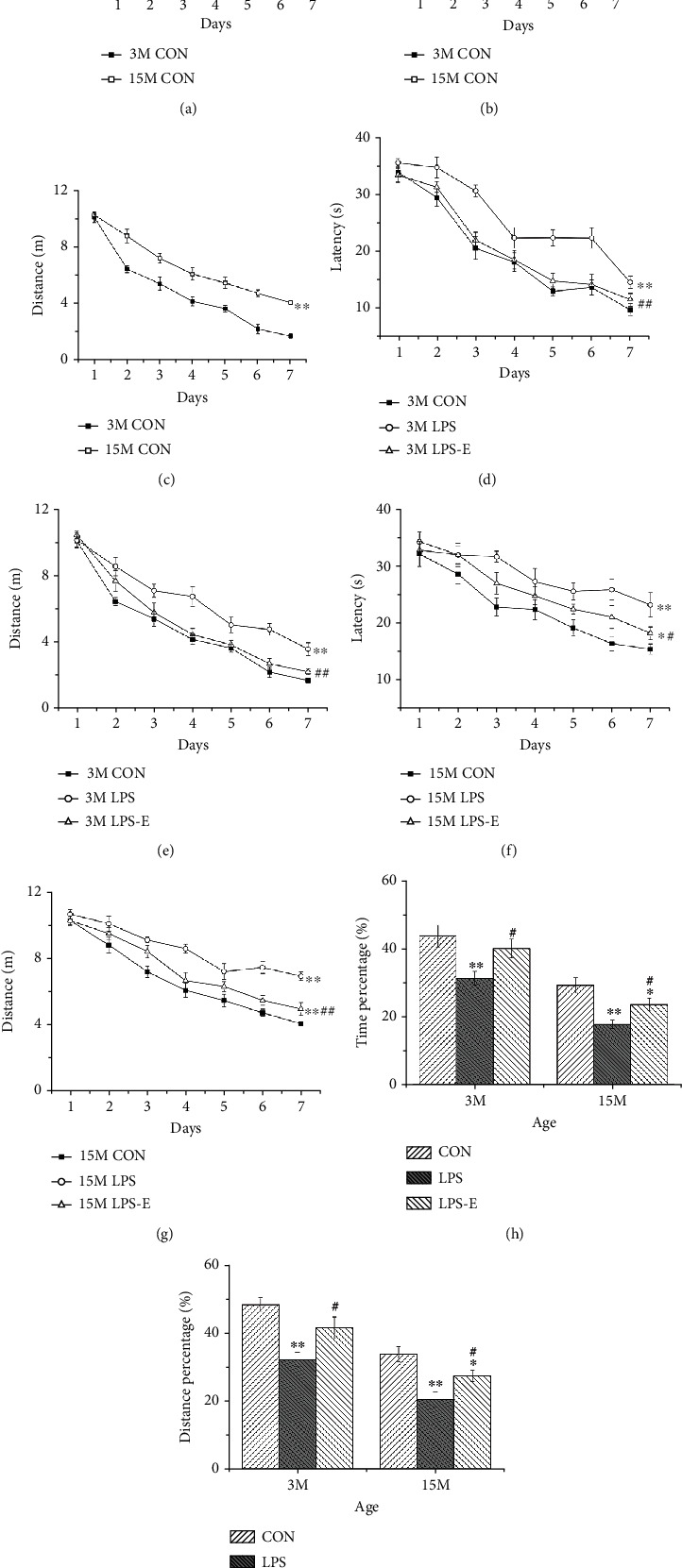
Learning and memory performance of in CD-1 mice in the MWM test. Latency (a), distance (b), and velocity (c) swam during the learning phase at different ages in the control groups. (d, e) Latency and distance during the learning phase by 3-month-old (3 M) and (f, g) 15-month-old (15 M) CD-1 mice in different treatment groups. The percent time (h) and distance (i) swam in the target quadrant in different treatment groups by 3 M and 15 M CD-1 mice. *n* = 10 per group. Error bars = SEM. ^∗^*P* < 0.05, ^∗∗^*P* < 0.01 compared with the control group; ^#^*P* < 0.05, ^##^*P* < 0.01 compared with the LPS group. CON: untreated control group; LPS: lipopolysaccharide treatment group; E: group of mice exposed to EE; MWM: Morris water maze.

Since the swimming latency and distance were affected by swimming velocity, we first analyzed velocity and found that this parameter was significantly affected by age. For example, at 15-months-of-age, mice had a significantly faster swimming velocity than at 3 months of age (*P* < 0.05, Figure 2(a)). Therefore, swimming latency and distance, particularly the latter, may be more reliable to evaluate the learning ability of mice than swimming speed.

#### 3.1.1. Age Effect

The swimming latency (*F*_(6,126)_ = 101.587, *P* < 0.01) and distance (*F*_(6,126)_ = 147.609, *P* < 0.01) decreased significantly for all mice with increasing time (days), thus indicating that these mice were able to learn in the MWM. Age significantly affected cognitive performance; at 15 months of age, mice had a significantly longer swim latency (*F*_(1, 14)_ = 7.739, *P* = 0.015) and swim distance (*F*_(1, 14)_ = 103.656; *P* < 0.01; Figures 2(b) and 2(c)), along with a higher percentage of swimming time (*t* = 3.667, *P* = 0.003) and distance (*t* = 4.769, *P* < 0.01) within the target quadrant than mice at 3 months-of-age (Figures 2(h) and 2(i)).

#### 3.1.2. Treatment Effect

During the learning phase, there were significant treatment effects on swimming latency and distance at 3 and 15 months of age (*F*_(2, 21)_ = 51.716, 25.868; *Ps* < 0.01). Post hoc analysis showed that the LPS group exhibited the worst performance according to both learning measures than the CON and LPS-E groups at both 3 and 15 months of age (*P* < 0.05 for all). However, the learning performance in the LPS-E was poorer than that in the CON group at 15 months of age (*P* = 0.024, 0.001, Figures 2(d)–2(g)). During the memory phase, the percentage of memory time and distance in the CON and LPS-E groups were significantly higher at both 3 and 15 months of age (*P* < 0.05 for all), when compared with the LPS group. At 15 months-of-age, the LPS-E group exhibited a significantly poorer memory performance than the CON group (*P* = 0.041, 0.038; Figures 2(h) and 2(i)).

### 3.2. Assessment of CORT, TNF-*α*, and IL-1*β* Levels in Serum

**Figure 3:**
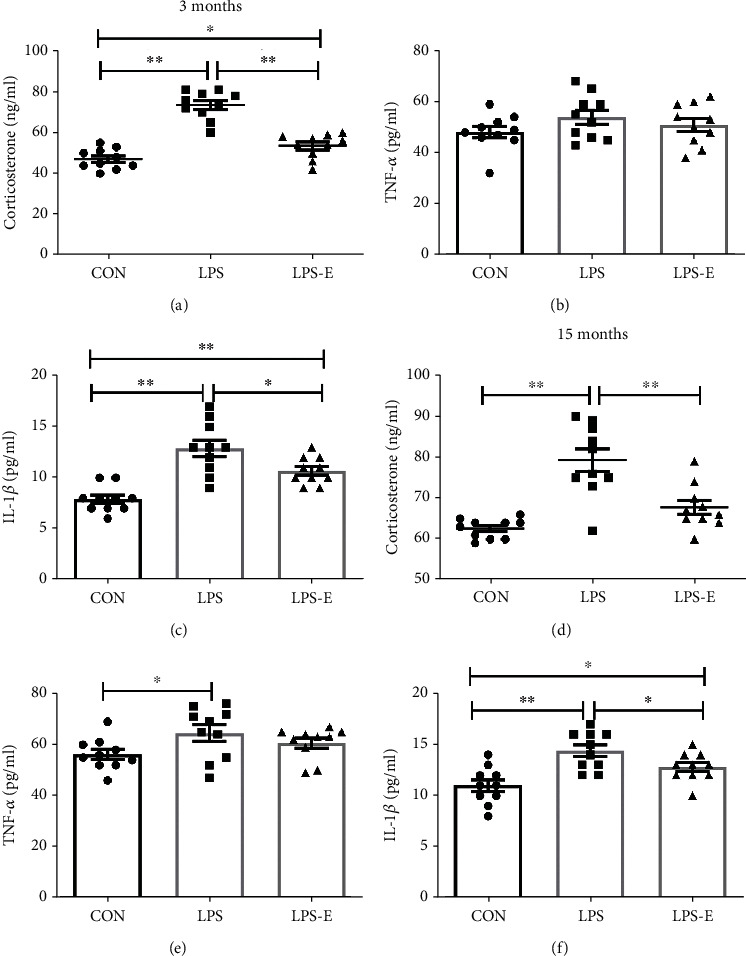
Plasma levels of corticosterone, TNF-*α*, and IL-1*β* levels in the CON, LPS, and LPS-E groups in offspring at 3 months (a)–(c) and 15 months (d)–(f). All data are depicted as mean ± SEM (*n* = 10 per group). Significance is as follows: ^∗^*P* < 0.05; ^∗∗^*P* < 0.01.

Next, we compared the serum levels of CORT, TNF-*α*, and IL-1*β* in mice from the CON, LPS, and LPS-E groups at 3 and 15 months of age (Figure 3). At 3 months of age, the LPS mice exhibited significantly higher serum levels of CORT than those in the CON group (*P* < 0.01). When considering the cytokine response to prenatal inflammation challenge, we observed a significant IL-1*β* response in the CON and LPS offspring; however, there was no response with regard to TNF-*α*. We found that the provision of an EE could alleviate the corticosterone and inflammatory cytokine responses, although the levels of CORT and IL-1*β* in the LPS-E group were significantly higher than those in the CON group (*P* < 0.05 for all); this may have been due to the insufficient duration of EE treatment (Figures 3(a)–3(c)). At 15 months of age, mice from the LPS group had higher serum levels of CORT, TNF-*α*, and IL-1*β* than those in the CON group (*P* < 0.05 for all); mice in the LPS-E group had lower levels of CORT and IL-1*β* than those in the LPS group (*P* < 0.05 for all). No differences were observed between the CON and LPS-E groups when considering CORT and TNF-*α* levels (*P* > 0.05 for all; Figures 3(d)–3(f)). These results indicated that chronic maternal inflammation could increase corticosterone levels and the cytokine response in offspring and that long periods of EE could ameliorate these adverse responses.

### 3.3. Increased Levels of Arc and Syt1 in the Hippocampus

**Figure 4:**
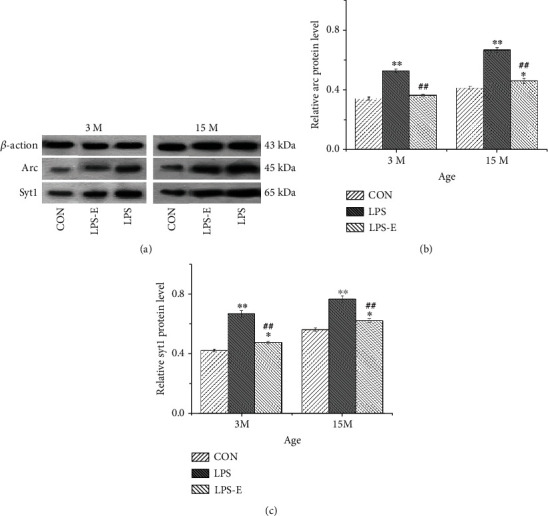
Representative immunoreactive bands for Arc and Syt1 (a) in the hippocampi of mice in different treatment groups at different ages. (b) Arc and (c) Syt1 protein levels in the hippocampi of mice at different ages in the different treatment groups (*n* = 6 per group). Error bars = SEM. ^∗^*P* < 0.05, ^∗∗^*P* < 0.01 compared with the control group; ^##^*P* < 0.01 compared with the LPS group. CON: untreated control group; LPS: lipopolysaccharide treatment group; E: group of mice exposed to EE.

Immunoreactive bands for synaptic proteins after western blotting are shown in Figure 4(a); at 15-months-of-age, mice had significantly higher levels of Arc and Syt1 proteins than at 3 months of age (*t* = −4.581, -9.199; *Ps* < 0.01), thus indicating that the levels of Arc and Syt1 protein increased with age. The LPS group exhibited the highest levels of Arc and Syt1 (*Ps* < 0.01) in both 3- and 15-month-old mice. Post hoc analysis showed that at 3 months, the LPS-E group exhibited significantly higher levels of Syt1 than mice in the CON group at 3 months of age (*P* < 0.05). At 15 months-of-age, the LPS-E group had lower (vs. the LPS group) or higher (vs. the CON group) levels of Arc and Syt1 (*Ps* < 0.05). Furthermore, the LPS-E group had higher levels of Arc and Syt1 than mice in the CON group (*Ps* < 0.05; Figures 4(b) and 4(c)).

### 3.4. mRNA Levels of *Arc* and *Syt1* in the Hippocampus

**Figure 5:**
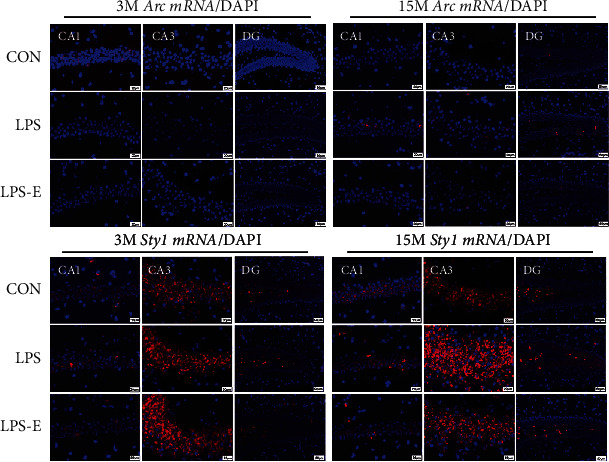
Representative photomicrographs of *Arc* and *Syt1* mRNA levels in different hippocampal subregions in CD-1 mice at different ages and under different treatments. Scale bar = 20 *μ*m in the CA1 and CA3, 50 *μ*m in the DG. CA: cornu ammonis; DG: dentate gyrus.

*In situ* hybridization fluorescent immunostaining showed that *Arc* and *Syt1* transcripts were primarily localized in the pyramidal cell layer (Figure 5).

**Figure 6:**
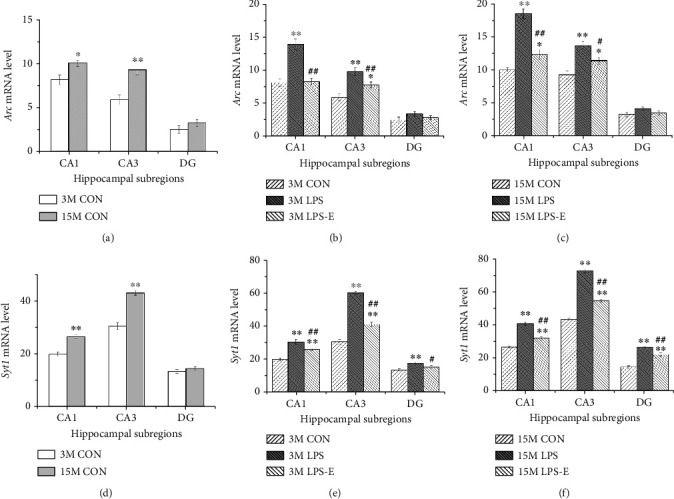
The expression levels of *Arc* and *Syt1* mRNA in different hippocampal subregions in CD-1 mice. (a) *Arc* mRNA and (d) *Syt1* mRNA levels in different hippocampal subregions (CA1, CA3, and DG) at different ages in the control groups. (b, c) *Arc* mRNA and (e, f) *Syt1* mRNA levels in different hippocampal subregions (CA1, CA3, and DG) in the different treatment groups (*n* = 6 per group) at 3 months of age (b, e) and 15 months of age (c, f). The relative mRNA level was calculated as the number of positive cells expressing mRNA. Error bars = SEM. ^∗^*P* < 0.05, ^∗∗^*P* < 0.01 compared with the control group; ^#^*P* < 0.05, ^##^*P* < 0.01 compared with the LPS group. CON: untreated control group; LPS: lipopolysaccharide treatment group; E: group of mice exposed to EE; MWM: Morris water maze.

In normal-aging mice, older mice exhibited higher levels of *Syt1* and *Arc* mRNAs in CA1 and CA3 than younger mice younger (*P* < 0.05 for all; Figures 6(a) and 6(d)). With regard to treatment effects, at 3 months of age, mice from the LPS group exhibited the highest levels of *Arc* and *Syt1* mRNAs in the CA1 and CA3 when compared to the other two groups (*P* < 0.05 for all). Furthermore, mice from the LPS-E group had significantly higher levels of *Arc* mRNA in CA3 than mice in the CON group (*P* = 0.022). Mice in the LPS group exhibited the highest levels of *Syt1* mRNA l in the DG than mice in the CON and LPS-E groups (*P* < 0.05 for all). At 15 months-of-age, mice in the AD LPS-E group had lower (vs. the LPS group) or higher (vs. the CON group) levels of *Syt1* and *Arc* mRNAs in CA1 and CA3 (*P* < 0.05 for all) and *Syt1* mRNA in the DG (*Ps* < 0.01; Figures 6(b), 6(c), 6(e), and 6(f)).

### 3.5. The Correlation between Cognitive Performance and Protein Levels

At 3 months of age, linear correlation analyses showed that the levels of Arc and Syt1 proteins in the LPS group were positively correlated with swimming latency and distance (*P* < 0.05 for all) and negatively correlated with memory time and distance (*P* < 0.05 for all). In addition, the levels of Syt1 were positively correlated with swimming latency and distance in mice from the LPS-E group (*P* = 0.026) and negatively correlated with the memory time and distance in the LPS group (*P* = 0.003; Table 1).

At 15 months of age, the levels of Arc and Syt1 in the LPS and LPS-E groups correlated positively with learning swimming latency and distance and were negatively correlated with the memory percentage of time and distance (*P* < 0.05 for all); similar findings were observed for the levels of Syt1 in the CON group (*P* < 0.05 for all). Furthermore, the levels of Arc protein in the CON group were negatively correlated with the memory percentage of time (*P* = 0.008; Table 1).

### 3.6. The Correlation between Cognitive Performance and mRNA Levels

At 3 months of age, the levels of *Arc* and *Syt1* mRNAs in CA1 and CA3 in the LPS group were positively correlated with swimming latency and distance and negatively correlated with the memory percentage of time and distance (*Ps* < 0.05). In addition, the levels of *Syt1* mRNA in CA1 in the LPS-E group were also positively correlated with swimming latency (*P* = 0.016) and negatively correlated with distance (*P* = 0.02). The levels of *Syt1* mRNA in CA3 in the LPS group were negatively correlated with the memory percentage of time and distance (*P* < 0.05 for all; Table 2).

At 15 months of age, the levels of *Arc* mRNA in CA3 and the levels of *Syt1* mRNA in CA1 and CA3 in the CON and LPS groups were positively correlated with latency and the distance swam (*Ps* < 0.05) and negatively correlated with the time and distance percentage in the target quadrant (*Ps* < 0.05). Moreover, the levels of *Arc* mRNA in CA1 and *Syt1* mRNA in CA3 in the LPS-E group were positively correlated with the latency and the distance swam (*P* < 0.05 for all) and negatively correlated with the time and distance percentage in the target quadrant (*P* < 0.05 for all). This was the same for the levels of *Syt1* mRNA in the DG in the LPS group (*P* < 0.05; Table 2).

## 4. Discussion

Infectious inflammation in the embryo may affect the development of the fetal nervous system and may interfere with the cognitive activity of the maternal and offspring, such as in the case of age-associated cognitive decline [13, 14]. It is well established that age-associated cognitive decline involves the disordered expression of proteins related to synaptic plasticity in the brain, particularly in the hippocampus [15, 16]. With regard to cognitive function, it has been consistently shown that the provision of an EE improves spatial learning in the Morris water maze [20, 30] and directly increases the number of synapses and dendritic spines [31–33] in the CA1 of the hippocampus. The expression of synaptic proteins in the hippocampus involves the AMPAR and N-methyl-D-aspartic acid (NMDA) receptor signaling pathway proteins, such as CaMKII, CREB, and Arc; these are all activated by exposure to EE. CaMKII and CREB are key downstream molecules in the NMDAR signaling pathway and play roles in long-term potentiation (LTP) consolidation and memory formation [34, 35]. Some studies have implicated both the AMPA and NMDA classes of glutamate receptors in synaptic change the following exposure to EE [36]. We were particularly interested in evaluating the effect of embryonic exposure inflammation on the expression of genes related to synaptic plasticity (*Arc* and *Syt1*) in mice and whether the provision of EE from adolescence could improve the expression levels of these genes. These changes may underlie the storage of spatial information associated with the enrichment stimulus.

First, we assessed corticosterone levels and inflammatory cytokine responses. Elevated corticosterone levels and cytokine responses were observed at both 3 and 15 months of age. Moreover, the provision EE alleviated these adverse responses, at least to some extent, especially in older mice (15 months of age) when exposed to long-term EE. The effects of EE in younger mice (3 months of age) receiving short-term exposure to EE were not as obvious. Whether corticosterone and cytokine responses contribute to age-associated cognitive decline in mice remains unclear.

In the present study, we demonstrated that CD-1 mice exposed to inflammation during embryonic life exhibited accelerated age-associated cognitive decline. More specifically, mice in the LPS and LPS-E groups, which experienced inflammatory challenge *in utero*, exhibited increased levels of synaptic proteins (Arc and Syt1) along with worse spatial learning and memory performance than mice in the CON group tested at 15 months of age (*P* < 0.05 for all). However, the behavioral performance of mice in the LPS-E group was significantly better than that of mice from the LPS group at both 3- and 15 months of age (*P* < 0.05 for all). This suggested that embryonic exposure to inflammation can accelerate age-associated cognitive decline, but also that the provision of EE (from adolescence) can improve age-associated cognitive decline. In terms of spatial learning and memory behaviors, as with our previous findings [14, 15, 28], we found that animals treated with LPS during the embryonic period exhibited a worse performance than those in the CON group as they aged. Furthermore, we found that there was an age-related increase in the expression of *Arc* and *Syt1* mRNA in the hippocampus. The levels of Arc and Syt1 proteins and mRNAs in the hippocampus of mice aged 15 months were significantly higher than those that were 3 months of age (*P* < 0.05). Furthermore, the expression levels of *Arc* and *Syt1* mRNA in the LPS group were significantly higher than those of mice in the CON and LPS-E groups in both the young and old groups (*P* < 0.05 for all). At 15 months of age, the expression levels of Arc and Syt1 proteins in the LPS-E group were significantly higher than those in the CON group, but significantly lower than those in the LPS group (*P* < 0.05 for all); we observed similar findings for the levels of *Arc* mRNA in CA3 and Syt1 mRNA in the CA1, CA3, and DG subregions of the hippocampus at 15 months-of-age (*P* < 0.05 for all). The expression levels of *Arc* and *Syt1* mRNA were significantly higher in the LPS group than in the LPS-E group at 3 and 15 months of age (*P* < 0.05) could improve but could not rescue the expression of *Arc* and *Syt1* mRNA in prenatal CD-1 mice exposed to inflammation. In addition, Pearson correlation analysis revealed that the expression of *Syt1* and *Arc* mRNA in the hippocampus was correlated with spatial learning and memory (*P* < 0.05 for all) (see Tables 1 and 2). We found that EE improved the inflammation-induced increase in Arc and Syt1 proteins and their mRNAs, thus suggesting that this complex condition acted as a positive stressor to protect the nervous system from inflammation. With regard to behavioral aspects, prenatal inflammation induced the overexpression of *Arc* and *Syt1* mRNA in the hippocampus and led to a series of spatial learning and memory deficits, including the acceleration of age-associated cognitive decline.

During development, Arc may modify the architecture of the hippocampal network via known effects on the homeostatic synaptic scale but also through hitherto unexplored mechanisms [37–39]. Moreover, Arc is bidirectionally regulated by NMDA and AMPA receptors; the relative extent of NMDA and AMPA receptor activation may be a critical determinant for the expression of *Arc* [40]. These data suggest the existence of a novel mechanism in which the pathways that control *Arc* transcription integrate signals from NMDA and AMPA receptors. Arc may also regulate the surface expression of AMPA receptors [41], and in turn, AMPA receptors, together with NMDA receptors, may regulate the expression of the *Arc* gene. Notably, inhibiting AMPA receptors strongly potentiated activity-dependent *Arc* expression [39]. The most novel aspect of the present study is our observation that prenatal inflammation increases the expression of *Arc* in the hippocampus. We hypothesize that this could result in an imbalance between Arc and AMPAR/NMDAR ratios due to aging or prenatal inflammation. This may also represent a compensatory brain mechanism for learning and memory consolidation.

Syt1 is a calcium sensor in the glutamatergic presynaptic terminals that regulate the SNARE-mediated exocytosis of neurotransmitter-containing vesicles [42, 43]. In a previous study, Hussain et al. demonstrated that this calcium sensor may also form a regulatory step for the insertion of AMPA receptors into the postsynaptic spine [44]. Modifying the synaptic expression of *Syt1* could be a possible mechanism to reduce the effects of postsynaptic calcium overload in excitotoxic diseases [45, 46]. Our current results showed that the levels of Syt1 protein and mRNA increased in the CA1 and CA3 subregions of the hippocampus with aging; these observations are consistent with our previous findings [15]. The long-term accumulation of Syt1 with age may have toxic effects on the nervous system, particularly on synaptic structure, thus exerting direct effects on synaptic transmission.

The intraperitoneal administration of LPS during gestation is known to not cross the placental barrier. However, direct placental damage and subsequent fetal damage caused by maternal LPS intervention have previously been shown to persist into adulthood [47]. Elevated levels of inflammatory cytokines in the fetal brain can also stimulate glial cells, thus resulting in lasting adverse effects on fetal growth, development, and postpartum neurobehavior; these cytokines can also lead to accelerated aging in the offspring. These processes may align with an age-related decline in synaptic function, such as damage being incurred by proteins related to synaptic plasticity, such as Arc and Syt1. Prenatally inflammatory insults could increase the hippocampal expression of *Arc* and *Syt1* mRNA in adult mice, as indicated by the higher levels of Arc and Syt1 proteins and mRNAs in the LPS mice when compared to mice in the CON group at both 3 and 15 months of age. The provision of an EE could relieve this effect, as indicated by the fact that levels of Arc and Syt1 proteins and mRNAs were lower in the LPS-E group relative to those in the LPS group. Furthermore, the dysregulation of proteins related to synaptic plasticity in the hippocampus induced by prenatal inflammation may represent one of the neurobiological mechanisms underlying age-associated cognitive decline. This study showed a link between synaptic plasticity-related genes *Arc* and *Syt1* expression and age-associated cognitive decline induced by prenatal inflammation combined with long-term EE. Furthermore, our findings add to the growing body of evidence that long-term environmental enrichment can ameliorate cognitive decline by altering the expression of synaptic plasticity-related genes. At the same time, unlike previous studies that focused on adult (6 months) and old age (18 months) [16], this study focuses on youth adult (3 months) and midlife (15 months), as well as other synaptic proteins (Arc/Syt1) in addition to Homer1/Glua1/PSD95. The two complement each other more effectively in proving the relationships between age-related cognitive decline and synaptic plasticity-related gene expression changes. However, we did not investigate the changes in synaptic plasticity in the hippocampal neurons. Future research should aim to identify evidence of electrophysiological changes in synaptic plasticity (such as LTP and LTD), as well as changes in other key proteins caused by EE. Another limitation of our present study was that we only detected gene and protein expression levels in males. Future research should investigate females and other age points.

## 5. Summary

Age-associated cognitive decline is a serious health concern in our aging society and involves the abnormal expression of synaptic proteins in the hippocampus. In this study, we found that prenatal inflammation led to the enhanced expression of *Arc* and *Syt1* mRNA in the hippocampus from a young age onwards, thus, leading to impairments in spatial learning and memory along with the process of normal aging. We demonstrated that exposure to an EE from adolescence could improve age-associated cognitive decline and facilitate spatial learning and memory by downregulating the expression of *Arc* and *Syt1* in the hippocampus.

## Figures and Tables

**Table 1:** Correlations between groups with regard to performance in the MWM and hippocampal synaptic proteins.

Cognitive phases	Ages	Groups	Synaptic proteins	
			Arc	Syt1
			*r* (*p*)	*r* (*p*)
Learning phase				
Swimming latency	3 months	CON	0.530 (0.177)	0.289 (0.488)
		LPS	0.815 (0.014)∗	0.797 (0.018)∗
		LPS-E	0.679 (0.064)	0.768 (0.026)∗
	15 months	CON	0.653 (0.079)	0.891 (0.003)∗∗
		LPS	0.851 (0.007)∗∗	0.755 (0.03)∗
		LPS-E	0.848 (0.008)∗∗	0.945 (0.000)∗∗
Swimming distance	3 months	CON	-0.176 (0.667)	-0.238 (0.571)
		LPS	0.907 (0.002)∗∗	0.905 (0.002)∗∗
		LPS-E	-0.267 (0.552)	-0.217 (0.606)
	15 months	CON	0.609 (0.109)	0.797 (0.018)∗
		LPS	0.951 (0.000)∗∗	0.959 (0.000)∗∗
		LPS-E	0.817 (0.013)∗	0.962 (0.000)∗∗

Memory phase				
Time percentage in target quadrant	3 months	CON	-0.140 (0.740)	0.086 (0.840)
		LPS	-0.712 (0.048)∗	-0.685 (0.061)
		LPS-E	-0.720 (0.044)∗	-0.557 (0.152)
	15 months	CON	-0.849 (0.008)∗∗	-0.849 (0.008)∗∗
		LPS	-0.940 (0.001)∗∗	-0.934 (0.001)∗∗
		LPS-E	-0.921 (0.001)∗∗	-0.935 (0.001)∗∗
Distance percentage in target quadrant	3 months	CON	-0.461 (0.250)	0.566 (0.143)
		LPS	-0.929 (0.001)∗∗	-0.889 (0.003)∗∗
		LPS-E	-0.744 (0.034)∗	-0.673 (0.068)
	15 months	CON	-0.611 (0.107)	-0.836 (0.01)∗
		LPS	-0.942 (0.000)∗∗	-0.829 (0.011)∗
		LPS-E	-0.744 (0.034)∗	-0.781 (0.022)∗

^∗^ *P* < 0.05; ^∗∗^*P* < 0.01.

**Table 2:** Correlations between groups with regard to performance in the MWM and hippocampal mRNAs.

Cognitive phases	Ages	Groups	*Arc* mRNA			*Syt1* mRNA		
			CA1	CA3	DG	CA1	CA3	DG
			*r* (*p*)	*r* (*p*)	*r* (*p*)	*r* (*p*)	*r* (*p*)	*r* (*p*)
Learning phase								
Swimming latency	3 months	CON	0.347 (0.399)	0.488 (0.220)	-0.278 (0.505)	0.363 (0.377)	0.175 (0.679)	0.405 (0.319)
		LPS	0.873 (0.005)∗∗	0.878 (0.004)∗∗	0.09 (0.833)	0.891 (0.003)∗∗	0.930 (0.001)∗∗	0.069 (0.870)
		LPS-E	0.177 (0.674)	0.394 (0.334)	-0.053 (0.901)	0.805 (0.016)∗	0.639 (0.088)	0.197 (0.640)
	15 months	CON	0.250 (0.550)	0.887 (0.003)∗∗	-0.115 (0.785)	0.909 (0.002)∗∗	0.858 (0.006)∗∗	-0.480 (0.228)
		LPS	0.496 (0.211)	0.813 (0.014)∗	-0.594 (0.120)	0.900 (0.002)∗∗	0.915 (0.001)∗∗	0.860 (0.006)∗∗
		LPS-E	0.727 (0.041)∗	0.672 (0.068)	-0.658 (0.076)	0.497 (0.211)	0.821 (0.012)∗	0.721 (0.044)∗
Swimming distance	3 months	CON	0.214 (0.611)	0.532 (0.175)	0.458 (0.254)	-0.200 (0.635)	-0.125 (0.769)	0.588 (0.125)
		LPS	0.877 (0.004)∗∗	0.848 (0.008)∗∗	0.089 (0.834)	0.877 (0.004)∗∗	0.736 (0.037)∗	-0.378 (0.356)
		LPS-E	0.128 (0.763)	-0.687 (0.060)	0.309 (0.457)	-0.232 (0.580)	-0.538 (0.169)	0.307 (0.459)
	15 months	CON	0.037 (0.930)	0.884 (0.004)∗∗	0.260 (0.534)	0.913 (0.002)∗∗	0.860 (0.006)∗∗	-0.614 (0.106)
		LPS	0.488 (0.265)	0.834 (0.010)∗	-0.558 (0.150)	0.917 (0.001)∗∗	0.858 (0.006)∗∗	0.821 (0.011)∗
		LPS-E	0.734 (0.038)∗	0.564 (0.145)	-0.528 (0.179)	0.669 (0.070)	0.809 (0.015)∗	0.801 (0.017)∗

Memory phase								
Time percentage in target quadrant	3 months	CON	0.566 (0.143)	-0.07 (0.869)	0.378 (0.356)	0.418 (0.303)	0.233 (0.579)	0.082 (0.848)
		LPS	-0.756 (0.03)∗	-0.797 (0.018)∗	0.185 (0.660)	-0.810 (0.015)∗	-0.816 (0.014)∗	0.321 (0.438)
		LPS-E	0.037 (0.931)	-0.124 (0.769)	-0.228 (0.490)	-0.600 (0.116)	-0.855 (0.007)∗∗	0.007 (0.987)
	15 months	CON	-0.394 (0.335)	-0.818 (0.013)∗	0.125 (0.767)	-0.899 (0.002)∗∗	-0.798 (0.018)∗	0.337 (0.414)
		LPS	-0.351 (0.393)	-0.933 (0.001)∗∗	0.577 (0.135)	-0.842 (0.009)∗∗	-0.781 (0.022)∗	-0.714 (0.047)∗
		LPS-E	-0.866 (0.005)∗∗	-0.679 (0.064)	0.603 (0.114)	-0.601 (0.115)	-0.789 (0.020)∗	-0.669 (0.070)
Distance percentage in target quadrant	3 months	CON	0.142 (0.738)	0.040 (0.924)	0.159 (0.706)	-0.01 (0.980)	0.372 (0.365)	-0.249 (0.552)
		LPS	-0.935 (0.001)∗∗	-0.947 (0.000)∗∗	-0.197 (0.640)	-0.921 (0.001)∗∗	-0.930 (0.001)∗∗	-0.203 (0.629)
		LPS-E	0.304 (0.464)	-0.447 (0.267)	-0.071 (0.867)	-0.788 (0.020)∗	-0.780 (0.022)∗	-0.035 (0.935)
	15 months	CON	-0.003 (0.994)	-0.888 (0.003)∗∗	-0.142 (0.738)	-0.902 (0.002)∗∗	-0.803 (0.016)∗	0.694 (0.056)
		LPS	-0.679 (0.064)	-0.733 (0.038)∗	0.352 (0.392)	-0.748 (0.033)∗	-0.786 (0.021)∗	-0.880 (0.004)∗∗
		LPS-E	-0.753 (0.031)∗	-0.468 (0.242)	0.276 (0.508)	-0.228 (0.587)	-0.818 (0.014)∗	-0.451 (0.262)

^∗^ *P* < 0.05; ^∗∗^*P* < 0.01.

## Data Availability

The data used to support the findings of this study are available from the corresponding author upon request.

## Abbreviations

MWM: Morris water maze
E/EE: Enriched environment
PSD-95: Postsynaptic density protein 95
AMPAR: *α*-Amino-3-hydroxy-5-methyl (-4-isoxazole propionic acid) type receptors
NMDAR: N-methyl-D-aspartic acid receptor
LTP: Long-term potentiation
LTD: Long-term depression
Arc: Activity-regulated cytoskeleton-associated protein
Syt1: Synaptotagmin-1
rm-ANOVAs: Repeated measures analysis of variance.
